# Forecasting the Impacts of Artificial Intelligence Assistance in Virtual Consultations for Chronic Obstructive Pulmonary Disease: Exploratory Futures Wheel Study

**DOI:** 10.2196/90208

**Published:** 2026-06-03

**Authors:** Pranavsingh Dhunnoo, Bertalan Meskó, Karen McGuigan, Vicky O’Rourke, Michael McCann

**Affiliations:** 1 Department of Computing, Atlantic Technological University, CoLab Building, ATU, Port Road, Letterkenny, County Donegal, F92 FC93, Ireland, 353 74 918 6000; 2 The Medical Futurist Institute, Budapest, Hungary; 3 School of Nursing and Midwifery, Queen's University Belfast, Belfast, Northern Ireland, United Kingdom; 4 Faculty of Business, Atlantic Technological University, Letterkenny, County Donegal, Ireland

**Keywords:** medical futures studies, Futures Wheel, foresight, futures studies, digital health, artificial intelligence, virtual consultation, remote care, telehealth, chronic conditions

## Abstract

**Background:**

While digital health technologies promise to reshape the medical journey, their potential might not be realized due to unforeseen implementation challenges. Notably, the future impact of artificial intelligence (AI) in virtual consultations has been poorly investigated.

**Objective:**

This study aims to explore, across 8 areas, the future impacts of a bespoke, co-designed AI tool for remote chronic obstructive pulmonary disease care from the perspectives of patients and health care professionals (HCPs) with the Futures Wheel (FW) method. It provides practical recommendations for conducting FW activities involving novel digital health tools.

**Methods:**

A pilot FW workshop was conducted with public and patient involvement members to gather feedback on the process. Subsequently, an exploratory, in-person FW workshop was conducted with 2 patients with chronic obstructive pulmonary disease and 2 HCPs who had previously been involved in the co-design of the bespoke AI tool. The central statement was as follows: “The bespoke AI tool is used in every virtual consultation.” Participants identified first- and second-order consequences across the following 8 areas of impact: HCP-patient relationship impact, psychological impact, social impact, educational impact, legal impact, ethical impact, health care delivery impact, and technology impact. Each participant discussed their individual input to provide additional context.

**Results:**

Regarding the HCP-patient relationship, patients foresee the tool’s impact as redefining the remote care dynamic with enhanced patient involvement, while HCPs identify its meaningful communication assistance. On the psychological impact, patients expect an enhanced level of empowerment and confidence, and HCPs anticipate improved understanding of patients’ emotional well-being with the AI tool’s assistance. As for social impacts, patients view the AI support as beneficial for social patient-HCP interactions, and HCPs foresee their workflow being enhanced with flexibility and collaboration. The AI’s educational impacts are expected to include, from patients’ perspectives, better familiarization of HCPs with individual patient cases and, from HCPs’ perspectives, improved support for training, upskilling, and administrative tasks. On the legal front, patients identify limited risks associated with the tool, and HCPs expect its features to lead to safer practices, contingent on regulatory compliance. Provided integrity and ethical use, the tool’s ethical impact is not perceived as significant by patients, while HCPs see its personalized features as leading to fair, individual remote assessments. Patients envision the AI tool’s impact on health care delivery as fostering patient-centricity, and HCPs anticipate strengthened remote care processes. Technologically, patients forecast a significant improvement to the current system, requiring adequate investment and resources, while HCPs expect complementarity between human input, AI, and the current system.

**Conclusions:**

The plausible AI-driven future of remote chronic care is a nuanced one. The FW method indicated that a bespoke, co-designed AI tool can positively support virtual care delivery and remote interactions while indicating potential risks. These insights can inform strategies related to early planning, governance, and implementation considerations.

## Introduction

In the 21st century, the health care sector has experienced dramatic changes with the advent of digital health technologies. The spectrum of such tools spans from hardware, for example, wearable sensors and robotics, to software such as blockchain and artificial intelligence (AI) [1]. The permeation of these technologies into the health care ecosystem has been reshaping, or even redefining, contemporary medical journeys for stakeholders within this landscape. For instance, patients can obtain clinical-grade, personalized health insights from wearables that they can use at home, while health care professionals (HCPs) can access AI assistance to support their decision-making [2].

While digital health technologies can improve the health care experience for both patients and HCPs, there remains the risk of such potential not being fully realized. In a sector as established as health care, the implementation of new mechanisms faces significant challenges, largely due to human factors [3]. Poorly designed tools that do not account for varying levels of digital literacy or usability can face poor adoption among patients [1]. From HCPs’ perspectives, they might not be motivated to use new technologies without clear advantages [1]. Overlooking human factors can limit technological breakthroughs in health care, underscoring the significance of the cultural element in digital health transformation [4]. In addition, there remain a number of potential challenges beyond the human element that can stifle effective digital health adoption. Factors including technical limitations, data integrity, and regulatory compliance represent possible barriers [3].

The successful implementation of digital health tools relies on adequate planning [3]. However, the novelty of such tools might not prepare stakeholders and health care systems to plan for their efficient adoption. The sparse use of such technologies in health care settings indicates a lack of evidence regarding their impact in practice. This can, in turn, negatively influence implementation and planning. There is a need to develop the ability to forecast possible futures to equip stakeholders with an anticipatory mindset or future thinking. Researchers have highlighted the value and need for implementing futures thinking in health care settings for meaningful planning [3,5].

There are several established methodologies for forecasting the future in the field of futures studies [5]. Such approaches can provide a deeper understanding of the impacts of upcoming trends and the prerequisites required to ensure their optimal and beneficial integration [6]. Furthermore, insights drawn from futures studies can inform decisions made by health care stakeholders in favor of desirable futures [6].

For the near future, virtual care and AI are considered health care megatrends [7], meaning trends that will have profound impacts on this particular sector. The valuable contributions of such technologically mediated health care approaches were highlighted during the COVID-19 crisis [2]. Kuziemsky et al [8] further identify a future with significant roles for AI in virtual care settings, from assisting in remote conversations and patient monitoring to guiding virtual assessments and evaluations. Despite the potential beneficial contributions of AI, there remains a risk in its deployment in health care settings. With the level of automation they bring about, they present the risk of automation bias, whereby users become overreliant on AI output, risking complacency and clinical robustness [9]. This can have severe downstream implications. A notable example comes from Obermeyer et al [10] study. The researchers identified racial bias in a common health AI model, where the algorithm less accurately detected the health risks of certain demographics. The recent rise in generative AI tools has surfaced another set of issues with AI technology. Of note is the effect of model “hallucination,” where incorrect or fabricated information is generated as plausible output [11]. Reliance on such AI results has, in some cases, led to severe patient harm. For example, a patient was subjected to bromine toxicity following an inaccurate recommendation from ChatGPT [12]. These examples highlight the risk of unequal or even harmful outcomes if the technology is applied uncritically. Furthermore, these technical and user failures present legal and ethical ambiguity on the subject of accountability. Depending on the use case, AI system, and jurisdiction, liability can fall on institutions, developers, or individuals [13]. This indicates the need for important considerations in the design of AI systems with a view toward future implementation and dedicated health care usage.

However, the future impact of AI in virtual consultations has been poorly investigated. In fact, despite the value of adopting futures studies in health care and medicine, and the need to develop a medical futures studies subfield, there remains a scarcity of such undertakings [5]. This represents a gap in the literature and, therefore, in our understanding. Considering the positive outcomes associated with remote care [14] and our earlier work on co-designing an evidence-based AI tool for remote chronic obstructive pulmonary disease (COPD) assistance [15,16], it is propitious to undertake a medical futures study to explore the future impact of such a tool and address the identified gap in our understanding. Such an undertaking aids in anticipating benefits, challenges, and requirements for its implementation in real-world clinical practice. It also addresses the limited number of futures studies in health care contexts.

We aimed to tackle this endeavor in this study. It describes the results of an exploratory medical futures study using the Futures Wheel (FW) method, aimed at gathering the insights of patients and HCPs on the plausible impact of having a bespoke AI tool used in virtual consultations for COPD care. The research question is as follows: “What are the potential future impacts of a bespoke AI tool used in virtual consultations for COPD from the perspectives of patients and healthcare professionals?”

Among the few medical futures studies that used FW, the focus was on eHealth [6], systemic impacts of COVID-19 [17-19], emerging infectious diseases [20], and the evolution of the health care sector [21]. Therefore, to the best of our knowledge, this study is the first FW undertaking to be publicly reported with a focus on AI and virtual consultations. As such, we also provide some reflections and practical suggestions for future research adopting the FW in a similar context.

## Methods

### The Futures Wheel Method

The FW method was devised by futurist Jerome C Glenn in 1971 [22] as a structured brainstorming method to map out the direct and indirect impacts of a central trend or event. It is an established participatory data collection method in futures studies, where a trend or event of interest is written at the center of a paper, and participants write their perceived first- and second-order impacts emerging from that central event. Glenn [23] further revised the FW in subsequent years to include a set of predetermined areas along which the central event’s impacts are to be considered. Version 2.0 of the FW can better organize discussions and various views on potential pathways that the central event may lead to.

Among futures methods, the FW is arguably the most accessible technique as it does not require any specialized tools. Considering the lack of familiarity with futures studies among the target participants, the accessibility of the FW was deemed adequate for this study. This methodological choice follows current recommendations for medical futures studies [24]. The FW is recommended for investigations aimed at exploring the consequences of a future event, as is the case in this study. In particular, the FW’s output lends itself to our aim of exploring and surfacing the ripple effects of implementing a bespoke AI tool in virtual consultations. In contrast, other foresight methods used in medical futures studies, such as Delphi studies, scenario planning, and policy analysis, are less accessible to participants or less relevant for our aims, as they can require specialized tools, prior familiarity with foresight techniques, or are beyond exploratory in scope [24]. Despite its simplicity and accessibility, the FW remains a very powerful method to explore the future [23]. Therefore, for the scope of this study, the FW was an appropriate choice.

**Figure 1.**
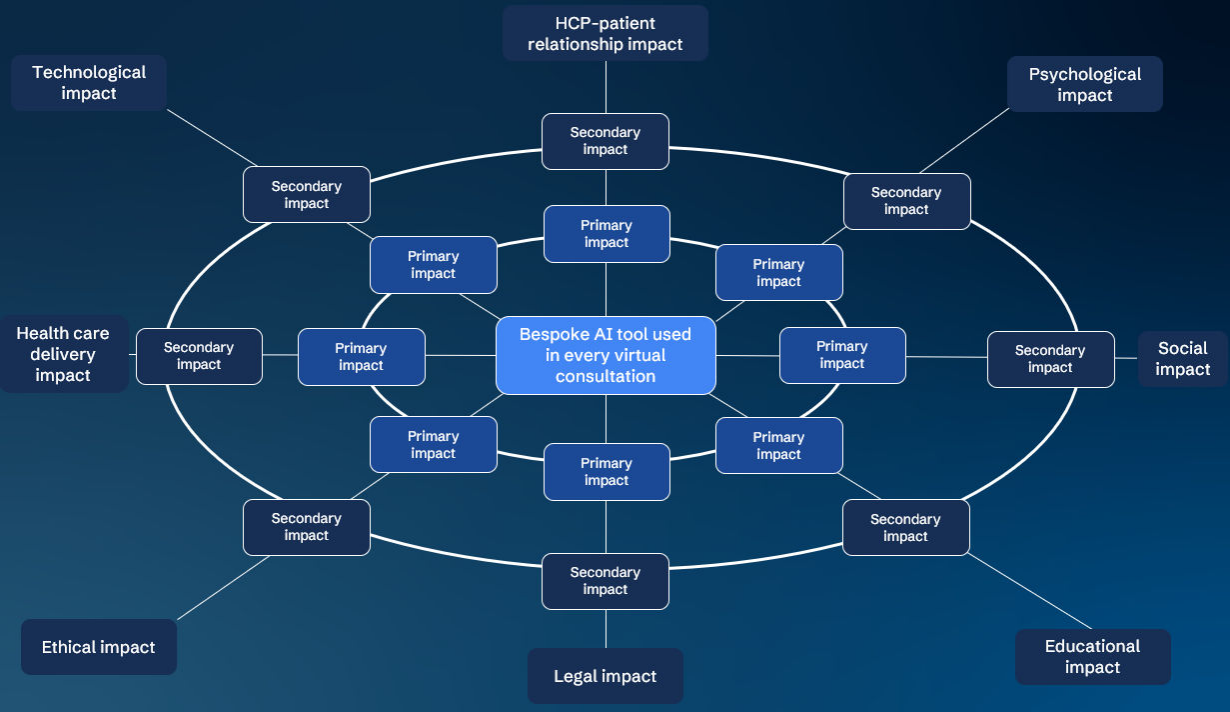
Futures Wheel template used in this study. AI: artificial intelligence; HCP: health care professional.

We conducted an in-person qualitative futures workshop using version 2.0 of the FW [23] with patients and HCPs who have experience in virtual consultations for COPD care. The central future statement was as follows: “The bespoke AI tool is used in every virtual consultation.” In introducing the updated FW version, Glenn [23] is not prescriptive in the predetermined areas of impact to guide discussions, but recommends that they be determined by the purposes of the analysis while remaining manageably broad. As such, the research team convened to discuss the impact areas to be included and agreed upon the following based on the areas of importance derived from findings of stages of the larger project of which this study is a part [14-16] and for their relevance to the participants: HCP-patient relationship impact, psychological impact, social impact, educational impact, legal impact, ethical impact, health care delivery impact, and technology impact. We acknowledge cross-cutting impacts in the chosen categories and that this may result in overlapping input. Glenn [23] acknowledges the potential for overlaps and even divergent perspectives from participants on the impacts due to the nature of the FW process. Glenn further argues that the ability to surface such convergence and divergence is a strength of the method. A protocol for this study was registered on Open Science Framework [25]. Figure 1 shows the FW template used in this study.

We adopted the SRQR (Standards for Reporting Qualitative Research) reporting guideline to draft this manuscript [26], and used the corresponding SRQR reporting checklist during the editing process (Checklist 1).

### Ethical Considerations

Considering the involvement of participants in conducting an FW investigation, the corresponding ethical considerations have been taken into account. Ethics approval was obtained from the Atlantic Technological University Research Ethics Committee (IREC-ATUD-23‐038) and the Letterkenny University Hospital Research Ethics Committee (24/221124). Participation was voluntary, and participants provided their fully informed, written consent prior to data collection. No compensation was provided for participation. To safeguard participant information, data were anonymized.

### Pilot and Data Collection Procedure

Prior to data collection, a pilot FW workshop was conducted with members of a public and patient involvement (PPI) committee to gather feedback on the process. PPI input is a recommended practice that supports and enhances study design [27]. The PPI committee members in this study were representative of the target participants, having experienced virtual consultations for chronic conditions. This allowed them to speak to the process for this cohort and provide valuable feedback.

During the actual FW workshop, the first author (PD) moderated and facilitated the activity with the participants. He did not contribute to the FW as a participant, but his role involved timekeeping, prompting discussion, and inviting critical appraisal. The workshop began with an explanation of the structured brainstorming process around the central event. Participants were provided with a printed version of the template (Figure 1), which they then filled out. Subsequently, each participant was invited to discuss their input in turn with the whole group, providing each participant with the opportunity to comment on and discuss their input on each segment of the FW, and even to challenge each other’s input. This approach adds to the quality control of the findings [28] and provides more context around their input. As such, the results are presented with corresponding quotes that illustrate the findings. A topic guide was used to facilitate the discussion (Multimedia Appendix 1).

The discussion was recorded via the General Data Protection Regulation–compliant Microsoft Teams software (Microsoft Corporation), and the automated transcription feature was used. PD reviewed the automated transcripts for accuracy, with corrections made to the text to ensure a verbatim transcript for analysis. Transcripts were anonymized, and the discussion provided added context to the findings. PD analyzed the FW input and discussions in each established segment to derive relevant insights as per the FW method [23]. We acknowledge that PD’s presence as moderator and facilitator potentially contributed to shaping group dynamics [29]. As such, BM provided rigor to the analysis by analyzing the data independently to confirm the relevance and accuracy of the data analysis. PD and BM convened to discuss and agree on the findings.

### Participant Sampling and Approach

All participants in this study had been involved in the co-design of the AI prototype, CARA (Consultation Analysis & Response Assistant), in our earlier study [15,16]. It is a browser-based tool that has dedicated patient- and HCP-facing interfaces. During the iterative co-design process, the participants had hands-on sessions with the tool and shared their feedback for improvements. This feedback was then integrated into improved versions of the tool. The close involvement of the participants in the co-design of the software endowed them with a deep understanding of the AI’s features. This familiarity with the tool is essential for envisioning and articulating its future impacts during the FW workshop.

Considering the novelty of the AI tool, its prototype status, and the practicalities of engaging participants in hands-on co-design sessions, an exploratory FW was deemed adequate for the scope of this study. For such undertakings, a single small group of 4 to 6 participants is recommended [30]. For this study, 4 participants (2 patients and 2 HCPs) were involved. We intentionally recruited individuals with experiential knowledge of the bespoke AI tool, considering the research focus on surfacing informed, context-specific, plausible impacts of that specific software’s use in virtual consultations. Among the few medical futures studies that used FW, those that reported their sample size included 9 [17], 10 [19], and 83 [21] participants, while the rest did not specify the number of participants involved [6,18,20]. This range of sample sizes (or lack of specificity thereof) is indicative of the nascency of medical futures undertakings, as well as the flexibility of the FW method. Notably, none of those former studies included a practical artifact with which participants required experiential knowledge. This further differentiates our exploratory study and its recruitment strategy, and we further reflect on this in “Recommendations for Future Research and Practice” and “Study Limitations” sections.

We emphasize that this study adopted an exploratory, qualitative foresight approach. Our pragmatic sample follows recommendations for “smart groups” size for exploratory FW that requires small groups of experts in a single workshop to explore the cascading effects from the central event [30]. The aim is thus to glean insights on the plausible future impacts of the bespoke AI tool, as well as on the FW exercise itself, which can guide subsequent work, as elaborated upon in “Recommendations for Future Research and Practice” section. Furthermore, specific, rare, and complex settings, as in the present case of bespoke AI-assisted remote care, often produce small samples [31]. The depth and contextual expertise of participants, combined with researcher reflexivity, can glean more important insights than larger projects with participants lacking experiential knowledge [32].

### Reflexivity

The research team acknowledges the potential influence of their individual backgrounds in guiding the analysis. The team is composed of an interdisciplinary group, with backgrounds in medicine and health care (PD, BM, KM), medical futures studies (PD, BM), marketing (VO), and engineering (MM). In line with best practice guidelines [33], the research team adopts an explicit reflexive stance that acknowledges that their individual experiences, backgrounds, and perceptions might influence the interpretation of the data, instead of assuming neutral observation.

To maintain analytic integrity during the analysis, complementary procedures were implemented, informed by best methodological practices. First, the inclusion of an interdisciplinary team aided toward balancing different perspectives in data collection and analysis. This contributes to making the researcher’s subjectivity more explicit [33]. There is also robustness in adopting reflexive guidelines [34,35], where the qualitative data were independently analyzed by the PD and BM who then discussed the results. This practice surfaces divergent readings and forces critical interrogation of analytic choices, all of which strengthen the reliability of the findings [36].

### Overview of the Features of CARA

To provide context for the AI tool designed to support virtual consultations between patients with COPD and their HCPs, we present an overview of its co-designed features in the following tables: Table 1 summarizes the features of the patient interface, and Table 2 summarizes the features of the HCP interface.

**Table 1.** Summary of AI^a^ features designed to assist patients on CARA’s^b^ dedicated patient interface.

Patient-facing feature	Description
Consultation analysis	Automated key takeaways and a simple summary of the consultation, along with automated clarifications of unfamiliar terms
Resource hub	Automatically suggests patient-oriented resources based on consultation context and provides the option to search for additional resources from a trusted health care institution’s database

a AI: artificial intelligence.

b CARA: Consultation Analysis & Response Assistant.

**Table 2.** Summary of AI^a^ features in CARA’s^b^ HCP^c^ interface to support remote interaction and clinical assessment.

HCP-facing feature	Description
Past consultation analysis	Automated insights from past consultations, including a clinically relevant summary, patient emotional status, and suggestions for upcoming consultations, in view of familiarizing HCP with individual patients.
Current consultation analysis section	Automated insights based on the current consultation, including a clinically relevant summary, clarification of unfamiliar terms, real-time analysis of the patient’s emotions, suggested next steps, and the option to generate a general practitioner referral letter.
Resource Hub	A querying tool that provides HCPs with resources drawn from a trusted health care institution’s database.

a AI: artificial intelligence.

b CARA: Consultation Analysis & Response Assistant.

c HCP: health care professional.

## Results

### Overview

Four participants from the Northwest region of Ireland were involved in the in-person FW workshop in August 2025. This included 2 male patients with COPD (aged between 65 and 75 y) and 2 female HCPs (1 advanced nurse practitioner aged between 55 and 60 y and 1 nurse aged between 35 and 40 y). All participants had experience with virtual consultations for COPD care in the Irish public health sector, and all were involved in the co-design of CARA.

During the workshop, which lasted for 47 minutes, the participants individually filled out the FW and discussed their input on each impact. PD moderated the discussion based on the topic guide (Multimedia Appendix 1), allowing each participant, in turn, to share their input on 1 FW segment before moving to the next segment. The participants shared their individual input in each FW segment with the whole group, and each participant could contribute to the discussion. As this study was interested in the perspectives of patients and HCPs, the respective insights are summarized in the separate FW diagrams below. This presentation of the results is a deliberate choice due to the inevitable difference in perspectives between patients and HCPs, given that the bespoke AI tool consists of dedicated patient and HCP interfaces that cater to their specific needs (Tables1  2).

**Figure 2.**
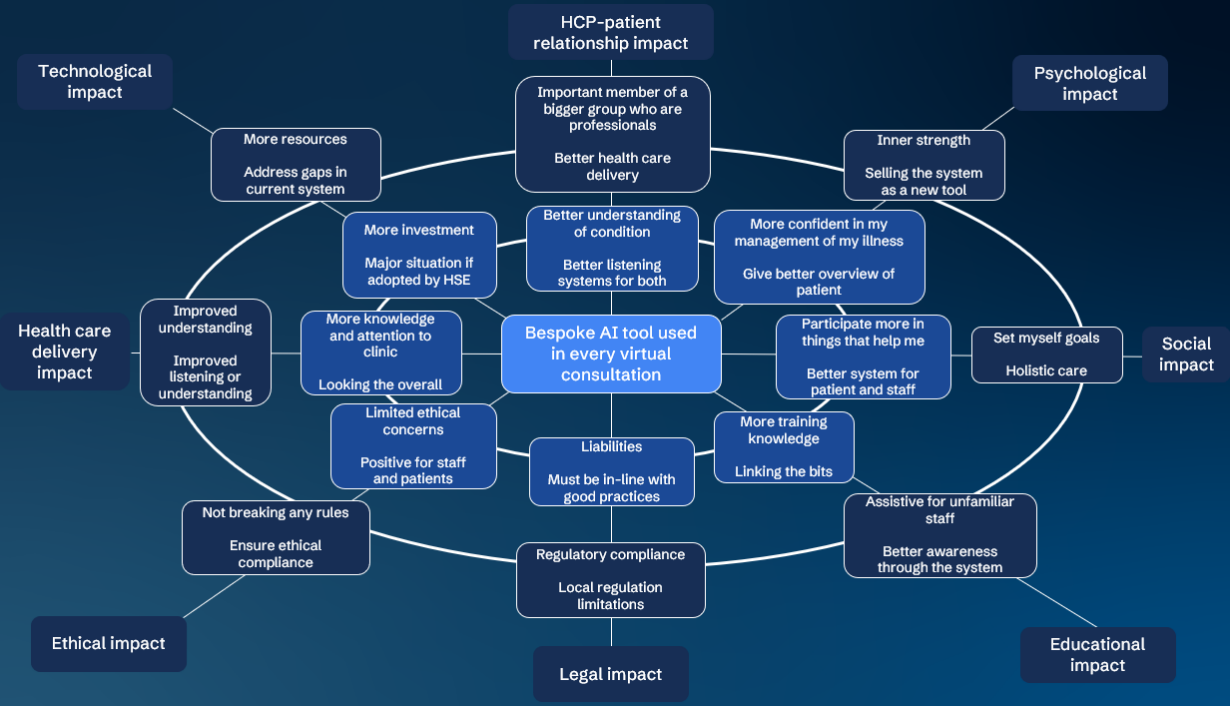
Futures Wheel results from the perspectives of patients with chronic obstructive pulmonary disease. AI: artificial intelligence; HCP: health care professional; HSE: health service executive.

Figure 2 summarizes the FW results from patients’ perspectives.

**Figure 3.**
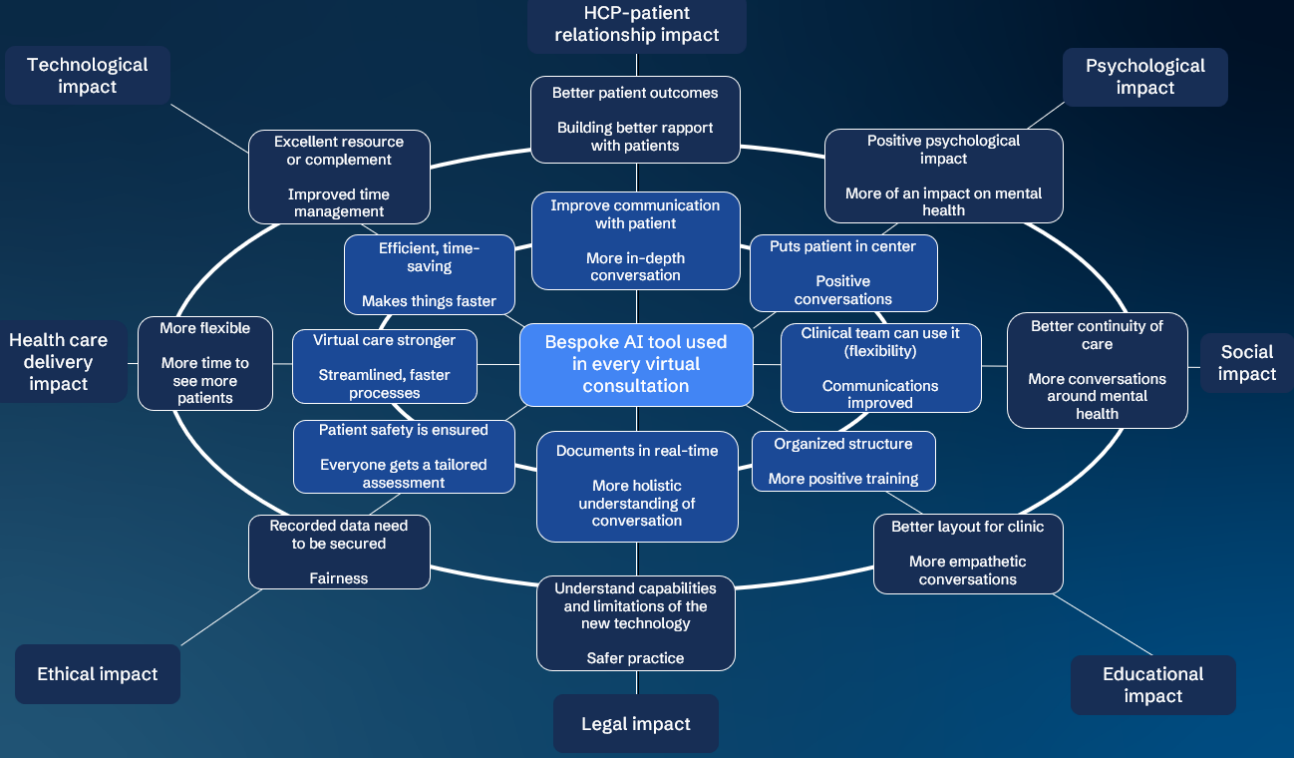
Futures Wheel results from the perspectives of HCPs attending patients with COPD remotely. AI: artificial intelligence; COPD: chronic obstructive pulmonary disease; HCP: health care professional.

Figure 3 summarizes the FW results from the perspective of HCPs.

The results of their input on each of the 8 impact areas are shared below, along with supportive quotes to illustrate their perspectives.

### HCP-Patient Relationship Impact

As a primary impact of the HCP-patient relationship, patients expect the tool to redefine the remote care dynamic. Drawing from its features that support conversations with summaries and context-relevant cues, it complements remote interactions with a more holistic view of their status. From their point of view, this contributes to a positive therapeutic relationship with enhanced patient involvement:

*[With CARA], there’s a better understanding of our conditions.*

[Patient #2]

*It’s not only the primary thing you're looking at. It’s the other thing. It’s like a holistic thing.*

[Patient #1]

*I would feel like an important member of a bigger group who are professionals.*

[Patient #2]

HCPs identified that the software would provide meaningful assistance in guiding remote communications if used in practice. As a result, this would lead to building better rapport with patients and enhancing patient outcomes:

*[There’s] more in-depth conversations [with CARA].*

[HCP #2]

*[It will lead to] better patient outcomes.*

[HCP #1]

### Psychological Impact

Having a tool such as CARA in practice appears to empower patients and has a positive psychological impact. The AI support is expected to build their confidence in managing their condition, thanks to the accessible insights it provides:

*It’s given me a lot more confidence actually managing my own condition.*

[Patient #2]

*It’s all explained like that, from a very simple structure that it’s not complicated, but it is complicated, but it’s simple at the end of the day.*

[Patient #1]

Personalized insights about the patient’s emotional well-being provided by the tool are expected to positively contribute to conversations initiated by HCPs. The resulting impact would aid in better understanding patients from a psychological point of view:

*Because we’re getting a better understanding that although I’m talking about respiratory conditions that they’re telling me, you know, it’s picking up on how they’re feeling.*

[HCP #2]

*Utilising the AI analysis of the consultation, it’s just all about creating a positive impact from a psychological perspective.*

[HCP #1]

### Social Impact

Patients view AI support in virtual consultations as beneficial for social patient-HCP interactions while encouraging self-management. Features such as automated patient resources and the ability for patients to find relevant health care resources through the software contribute to this sentiment:

*[CARA’s a] better system for patient and staff.*

[Patient #1]

*From all [of this support], you can set yourself goals.*

[Patient #2]

For HCPs, having the tool implemented in practice would, as a primary social impact, supplement the clinical workflow with added flexibility and collaboration. This likely draws from CARA offering assistance, such as generating clinically relevant summaries with actionable insights that aid in familiarizing with patients. The shared insights would thus lead to better care, with regard to the mental well-being component of patients:

*So I think it moves from just from one clinician to a team. The team can use this [insight].*

[HCP #1]

*There are more conversations around mental health, making you more aware of conversations that you're having.*

[HCP #2]

### Educational Impact

With the automated, personalized consultation summaries, patients view the tool as addressing current limitations in familiarizing and educating HCPs about individual patient cases. As a result, this can lead to better clinical workflows and training among new staff:

*It provides a bit more for people coming on to train, maybe new nurses or whatever.*

[Patient #2]

*Everyone has a better outline from day one when they start, both patient and the staff, and the clinicians.*

[Patient #1]

On the educational front, HCPs identified a dimension of training and upskilling for students and staff with the automated AI output. These are also expected to support staff with administrative tasks. Features of CARA, such as automating referral letters and providing a holistic overview of patients’ statuses, are relevant to these forecasts:

*The way you recap the appointment, it’s a better layout for the clinic, and with the summary, it would support education too.*

[HCP #1]

*For student nurses, [with CARA, there’ll be] more positive training.*

[HCP #2]

### Legal Impact

While patients identify limited risks with CARA from a legal perspective, they highlight that the tool needs to comply with the corresponding regulations. As a result, there might be a need to update local regulations to account for the use of this type of AI assistance in practice:

*I don't see anything that for me would be liable or something.*

[Patient #2]

*I know the HSE wants to be more in line with good practice across the EU.*

[Patient #1]

*I think it has to begin [...] to see where your work is legally and move forward.*

[Patient #1]

With its real-time documentation and support for improved communication, HCPs believe that CARA’s automated features will lead to safer practices. This would be contingent on its compliance with regulations:

*It documents in real time, so that’s quite good from a legal perspective.*

[HCP #1]

*You're picking up on all the things that you wouldn't have been picking up before, and that would lead to safer practice.*

[HCP #2]

*It’s new, and we need to make sure the legal aspect was tight.*

[HCP #1]

### Ethical Impact

Patients did not identify significant ethical concerns with having such automated assistance. They viewed it as having a positive impact on remote care practices, as long as its use remains ethical:

*I've listened to a whole lot of arguments, and I totally agree with you. We don't have a problem with it*.

[Patient #1]

*I don't think there’s anything there that’s breaking any rules.*

[Patient #2]

*I don't see why they would have a problem with it.*

[Patient #2]

From HCPs’ perspectives, CARA’s personalized features would lead to fair, individual remote assessment. They also highlight that the integrity of patient data must not be overlooked when it is implemented:

*It does a bit of background, and things like that. So it’s picking up on everything.*

[HCP #2]

*So that the ethics in and around taking this data and managing that data [needs to be secured].*

[HCP #1]

### Health Care Delivery Impact

From the patients’ perspective, they envision the software to enhance the remote care process with a focus on patients while supporting them in better understanding their condition. CARA’s HCP-facing features, such as insights into their past consultations and emotional status, along with its patient-facing features that provide accessible resources, likely contribute to these expectations.

*I think that’s a positive to me, [with improved] listening and understanding, uhm, [during] a meeting between a patient and a clinician*.

[Patient #1]

*I think with this here, I can make myself more knowledgeable. I can ask questions.*

[Patient #2]

CARA’s features are seen as an upgrade to health care delivery from the HCPs’ point of view. It would strengthen remote care with faster processes. As a result, remote care would become more flexible and allow HCPs to spend more time with patients:

*So you would see more patients, and that it would be streamlined.*

[HCP #2]

*I thought the virtual care would be stronger than the current practice, and I thought it would be more flexible.*

[HCP #1]

### Technology Impact

If the AI tool is implemented in practice, patients believe that it will be a significant improvement to the current system. They acknowledge the need for adequate investment and resources but view it as worthwhile, considering the benefits to patients and the system as a whole:

*Just imagine what better care they'd be able to give [with CARA]*.

[Patient #1]

*It'll mean everything needs more investment, more resources.*

[Patient #2]

*It is not a huge investment for the HSE, but it’s a huge investment for patients and their care.*

[Patient #1]

Having access to CARA in practice is viewed as an efficient collaboration between humans and AI. With its automated features, it saves time for HCPs and complements the current system:

*[CARA] makes things faster. For example, generating letters.*

[HCP #2]

*I thought it would be time-saving, so it would be an efficiency. And it would be an excellent complement [to our system].*

[HCP #1]

## Discussion

### Principal Findings

This exploratory medical futures study aimed to gather insights on the future impact of integrating a bespoke, co-designed assistive AI tool in virtual consultations for COPD. The focus was on the perspectives of patients and HCPs regarding specific areas of impact. The findings of the FW exercise provided nuanced insights from the respective cohorts’ visions of how integrating the bespoke tool into practice would reshape their virtual consultation experiences. Therefore, such FWs could provide policymakers with a clear panel of insights about developing and implementing a particular technology. As such, the method used in this study and the findings provide important contributions.

To the best of our knowledge, this is the first undertaking that uses the FW method to investigate the future impact of AI in virtual consultations. This section interprets these findings in relation to the extant literature and draws implications for future research and practice.

### Impact of AI in Virtual Consultations for COPD Care

Participants in this study foresee a reimagined therapeutic relationship in remote care with the assistance of CARA. This sentiment is shared among both patients and HCPs (“HCP-Patient Relationship Impact” section) and likely draws from its purpose-built features aimed at improving communication and enhancing rapport building (Tables1  2). This highlights the suboptimal quality of interactions in current virtual consultations [15], which negatively impact the overall quality of care and the therapeutic relationship in remote settings. HCPs even anticipate that the tool’s insights into patients’ emotional well-being will positively contribute to conversations, enabling a more holistic understanding of patients (“HCP-Patient Relationship Impact” section). The application of AI in clinical settings has raised concerns about the loss of the human touch [37]. However, it appears that if built to support interactions rather than replace them, as is the case with our co-designed prototype, AI assistance is perceived as positively reinforcing the patient-HCP relationship. In a future of remote care supported by AI, it could therefore be recommended that systems encourage the integration and investment in automated solutions that enhance, rather than diminish, the therapeutic relationship and the human touch.

The redefined remote care dynamic is further supported by the positive psychological support perceived, especially by patients (“Psychological Impact” section). Chronic conditions can have a significant impact on the mental well-being of patients living with such conditions [38], and they are likely to require psychological support mechanisms. It is notable that they not only find CARA’s assistance to be a tool for information about their condition but also a source of emotional reassurance that builds their self-confidence and makes them more engaged in managing their condition (“Psychological Impact” and “Social Impact” sections). This indicates a potential lack of such support in current COPD care, and empowering such patients has been associated with improved outcomes [39]. AI’s potential to empower patients has been highlighted in recent studies [40], and our findings indicate that the bespoke tool seems to have a similar effect. It could further be recommended for remote systems to include an element of psychological support and patient empowerment, whether through automated or traditional means, in view of contributing to better outcomes.

From HCPs’ perspectives, the automated assistance is considered a mechanism to add efficiency to their workflow (“Social Impact,” “Educational Impact,” “Health Care Delivery Impact,” and “Technology Impact” sections). They expect the bespoke tool to support flexible collaboration among colleagues while also supporting their upskilling and training. There is an underlying implication that the current remote care workflow might not be optimal and presents a learning curve, and automated tasks and patient insights can assist in this regard. Patients also believe that the AI’s features support the onboarding of trainees and new staff, thereby addressing current limitations and leading to better levels of care delivery (“Educational Impact” and “Health Care Delivery Impact” sections). Such an observation likely draws from past experiences with staff unequipped to adapt their interactions in virtual settings [41]. As such, participants foresee CARA streamlining remote care delivery with added efficiency and flexibility while offering fair, patient-centered care. This suggests that a hybrid human-AI collaboration, where algorithms meaningfully complement HCP tasks and needs, can contribute to favorable perceptions of such tools and lead to better rates of adoption among the medical community. Complementarity has been identified as a central factor for adoption among medical professionals [42], and having such an element in AI systems can potentially lead to more effective integration. On the practical side, in planning for future remote care systems, it might also be appropriate to train the workforce in optimizing remote interactions and workflows.

Overall, the findings indicate a plausible future with generally positive impacts of AI in virtual consultations across 8 areas of focus. It should be noted that this observation applies to CARA, a bespoke, co-designed tool, rather than other AI software. This bespoke tool was designed in an evidence-based manner with the close involvement of end users to reflect their needs and preferences [15]. While the findings are limited to our cohort of participants, participatory approaches in developing digital health tools such as CARA can potentially yield better perceptions and acceptance of novel tools. This aligns with reflections and recommendations from existing literature [43-45].

Nevertheless, participants cautioned about the need to ensure data integrity and regulatory compliance when implementing such novel tools in practice, despite their positive contributions to health care delivery (“Legal Impact” and “Ethical Impact” sections). From the patients’ standpoint, as CARA increases access to patient resources and self-directed care, they identify limited ethico-legal risks, but their reflections implicitly indicate the need to ensure equity and data security. HCPs emphasized the need for safeguards to ensure regulatory and data compliance. These are prevalent concerns among patients concerning AI in health care [46,47], which can potentially be addressed with human-centered design of such tools along regulatory frameworks from the planning stages. Notably, the limited ethical and legal risks that the participants identified suggest a potential limitation in the thoroughness of their understanding of the breadth of AI’s impact. As discussed earlier in the “Introduction” section, AI implementation in health care contexts presents a range of risks from automation bias [9] and real patient harm [10,12] to ethical and legal challenges [13]. While it is possible that participant’s concerns over these areas of impact might have been assuaged by their direct involvement in CARA’s co-design and their understanding of its assistance, embedded human oversight, and limitations, there is also the possibility of “unrealistic optimism” [48]. This refers to the tendency of people to not expect themselves to be victims of adverse effects; in this case, due to not being fully cognizant of AI’s complexities, likely due to CARA’s novelty. This observation derived from the FW insights thus indicates the need for careful implementation strategies around AI tools, possibly including an element of enhanced AI literacy, even if their assistance is evidence-based. This further indicates the need to include other stakeholders, such as policymakers and hospital managers, in designing such products to ensure their compliance in real-world practice.

### Recommendations for Future Research and Practice

The findings of this study have provided crucial insights for future research and practice. As the current scope was at an exploratory level, future research could expand participation with additional stakeholders, such as policymakers, developers, and hospital managers in larger groups to surface a broader range of perspectives and considerations that would enrich the diversity and complexity of the identified impacts. They could integrate longer or additional workshops to further explore more complex and interconnected impacts. In line with medical futures guidelines [24], such an approach could expand the gathered foresight insights to develop actionable implementation strategies.

For instance, Figures 1 and 2 can offer a preliminary roadmap for discussions with the Irish Health Service Executive, as the participants involved in this study draw their virtual consultation experiences from the health service executive. The findings are highly relevant to this context, and discussions could center around the patient and HCP perceptions of such AI assistance in future practice.

The findings of our FW can already be used to anticipate issues and challenges, such as equity and data protection concerns, which surfaced during our analysis. As the AI prototype is at a functional level that can be piloted in low-risk virtual consultations, subsequent studies involving CARA in real-life pilots could address such challenges ahead of involving participants. Considering the generally positive perspectives on the bespoke AI’s impact on remote care practices, there is value in considering the tool as a candidate for such studies to further assess its contribution in practical scenarios.

On a practical level, for conducting FW studies with digital health technologies, based on the authors’ experience, it is recommended that participants familiarize themselves with the tools, ideally through hands-on sessions. As our FW study followed co-design sessions where participants used the tool in question, this facilitated discussions around areas of impact of interest during their forecasting exercise. This might limit the number of participants due to logistical considerations, but we argue that the findings would be stronger as they are more contextual.

We also highly recommend piloting FW or other medical futures methods prior to data collection, preferably with a PPI committee. In our experience, their input helped improve the discussion elements as well as the format in which the study was conducted. For instance, since participants needed to familiarize themselves with the features of the AI prototype, which required guidance from PD, hosting the workshop in person was deemed optimal following the PPI pilot.

### Study Limitations

While our study has followed recommended practices and guidelines [5,24] and uncovered valuable insights, especially considering the scarcity of similar investigations, we acknowledge its limitations. Notably, the number of participants indicates the need for caution when interpreting the findings. While such small groups are adequate for exploratory FW studies [30], they can limit the depth of insights drawn. However, on a practical level, small groups are more manageable, especially if they are to have hands-on sessions with new tools. Future research that expands beyond an exploratory level could address this limitation by organizing multiple workshops with small groups and collating their insights. Therefore, the current output of this FW undertaking is more exploratory than confirmatory, in line with its scope.

The participant demographics involved in this study also limit its generalizability. While limited to the perspectives of the public health care system in the Northwest of Ireland and COPD as a condition, our study offers valuable academic contributions regarding the potential impact of such a bespoke AI tool in future practice, as it has provided practical insights that can inform future research and practice.

Another limitation of the sampling strategy is the potential for selection bias. Since all participants were involved in the AI tool’s co-design, they might have tended to view its impact more positively and might have been less likely to identify its risks. Future studies that go beyond an exploratory scope could conduct an FW activity with participants who were not involved in the co-design. However, there is value in including participants with experiential knowledge at this stage, as it can surface crucial, context-relevant insights that would not be possible with participants lacking familiarity with the tool [49].

Due to the inherent uncertainties in contemplating the future and the subjective influences of participants’ perceptions [24], the FW forecasts do not guarantee the likelihood of any particular outcome. Nevertheless, the findings provide constructive foresight that can assist in decision-making toward desirable outcomes, especially toward early planning, governance, and implementation considerations.

### Conclusions

There is a plausible, long-term future where AI becomes an integral component of remote chronic care. Contemplating such a future with patients and health care professionals indicates the practical benefits of embedding such automated assistance. They perceive positive contributions not only from technological and operational levels but also on the level of the therapeutic relationship and patient empowerment. Their articulated forecasts indicate a future that is hopeful yet cautious. In that desirable future, with a bespoke co-designed tool, there is complementarity between humans and AI that enhances the interaction quality, and successful implementation is supported by strong governance. This study has uncovered crucial insights that can guide stakeholders and medical futures practitioners, as well as academic and practical endeavors in navigating the uncertain future of AI-driven care.

## Supplementary material

10.2196/90208 Multimedia Appendix 1 Futures Wheel topic guide.

10.2196/90208 Checklist 1 SRQR checklist.

## Abbreviations

AI: artificial intelligence
CARA: Consultation Analysis & Response Assistant
FW: Futures Wheel
HCP: health care professional
PPI: public and patient involvement
SRQR: Standards for Reporting Qualitative Research

## References

[1] Yeung AWK, Torkamani A, Butte AJ (2023). The promise of digital healthcare technologies. Front Public Health.

[2] Han JH, Lee JY Digital healthcare industry and technology trends.

[3] Thimbleby H (2013). Technology and the future of healthcare. J Public Health Res.

[4] Meskó B, Drobni Z, Bényei É, Gergely B, Győrffy Z (2017). Digital health is a cultural transformation of traditional healthcare. mHealth.

[5] Meskó B, Kristóf T, Dhunnoo P, Árvai N, Katonai G (2024). Exploring the need for medical futures studies: insights from a scoping review of health care foresight. J Med Internet Res.

[6] Kouri P, Hopia H, Hakala A (2020). Predicting the future of healthcare and eHealth with the Futures Wheel method. J Int Soc Telemed eHealth.

[7] Deml MJ, Jungo KT, Maessen M, Martani A, Ulyte A (2022). Megatrends in healthcare: review for the Swiss National Science Foundation’s National Research Programme 74 (NRP74) “smarter health care”. Public Health Rev.

[8] Kuziemsky C, Maeder AJ, John O (2019). Role of artificial intelligence within the telehealth domain. Yearb Med Inform.

[9] Skitka LJ, Mosier KL, Burdick M (1999). Does automation bias decision-making?. Int J Hum Comput Stud.

[10] Obermeyer Z, Powers B, Vogeli C, Mullainathan S (2019). Dissecting racial bias in an algorithm used to manage the health of populations. Science.

[11] Kim Y, Jeong H, Chen S (2025). Medical hallucinations in foundation models and their impact on healthcare. arXiv.

[12] Eichenberger A, Thielke S, Van Buskirk A (2025). A case of bromism influenced by use of artificial intelligence. Ann Intern Med Clin Cases.

[13] Bottomley D, Thaldar D (2023). Liability for harm caused by AI in healthcare: an overview of the core legal concepts. Front Pharmacol.

[14] Dhunnoo P, Kemp B, McGuigan K, Meskó B, O’Rourke V, McCann M (2024). Evaluation of telemedicine consultations using health outcomes and user attitudes and experiences: scoping review. J Med Internet Res.

[15] Dhunnoo P, McGuigan K, O’Rourke V, Meskó B, McCann M (2026). User perceptions of virtual consultations and artificial intelligence assistance: a mixed methods study. Future Internet.

[16] Dhunnoo P, Meskó B, O’Rourke V, McGuigan K, McCann M, Arai K (2024). Intelligent Systems and Applications.

[17] Nouhi M, Heydari M, Goudarzi Z, Shahtaheri RS, Ahmadzaeh A, Olyaeemanesh A (2022). The future effects of COVID-19 on the health system: applying the Futures Wheel method. Med J Islam Repub Iran.

[18] Daffara P (2020). Applying the Futures Wheel and macrohistory to the COVID-19 global pandemic. J Futures Stud.

[19] Rezaei H, Haghdoost A, Javar HA (2021). The effect of coronavirus (COVID-19) pandemic on medical sciences education in Iran. J Educ Health Promot.

[20] Nejatzadehgan Eidgahi Z, Dehnavieh R, Borhaninejad V (2024). The Futures Wheel model of the effects of the emerging infectious diseases pandemics on the elderly in Iran. Salmand.

[21] Hopia H, Hakala A (2015). Finnish social and health care professionals’ perspective of the future. Int J Healthcare.

[22] Glenn J (1972). Futurizing teaching vs. futures course. Soc Sci Rec.

[23] Glenn JC, Glenn JC, Gordon TJ (2009). Futures Research Methodology.

[24] Mesko B, Kristóf T, Dhunnoo P, Árvai N, Katonai G (2025). A practical guide to using futures methods in health care: approaches, applications, and case studies. J Med Internet Res.

[25] Dhunnoo P, Meskó B, O’Rourke V (2025). Protocol for the co-design and medical futures studies of an assistive artificial intelligence tool for remote chronic care. Open Science Framework.

[26] O’Brien BC, Harris IB, Beckman TJ, Reed DA, Cook DA (2014). Standards for reporting qualitative research: a synthesis of recommendations. Acad Med.

[27] Coupe N, Mathieson A (2020). Patient and public involvement in doctoral research: impact, resources and recommendations. Health Expect.

[28] Nielsen AF, Michelmann J, Akac A (2023). Using the Futures Wheel methodology to assess the impact of Open Science in the transport sector. Sci Rep.

[29] Ozkaynak M, Sircar CM, Frye O, Valdez RS (2021). A systematic review of design workshops for health information technologies. Informatics.

[30] Bengston DN (2016). The Futures Wheel: a method for exploring the implications of social–ecological change. Soc Nat Resour.

[31] McDermott R (2023). On the scientific study of small samples: challenges confronting quantitative and qualitative methodologies. Leadersh Q.

[32] Crouch M, McKenzie H (2006). The logic of small samples in interview-based qualitative research. Soc Sci Inf.

[33] Finlay L (2002). “Outing” the researcher: the provenance, process, and practice of reflexivity. Qual Health Res.

[34] Braun V, Clarke V (2006). Using thematic analysis in psychology. Qual Res Psychol.

[35] Braun V, Clarke V, Hayfield N, Davey L, Jenkinson E, McBeath A, Bager-Charleson S (2023). Supporting Research in Counselling and Psychotherapy: Qualitative, Quantitative, and Mixed Methods Research.

[36] Nowell LS, Norris JM, White DE, Moules NJ (2017). Thematic analysis: striving to meet the trustworthiness criteria. Int J Qual Methods.

[37] Witkowski K, Dougherty RB, Neely SR (2024). Public perceptions of artificial intelligence in healthcare: ethical concerns and opportunities for patient-centered care. BMC Med Ethics.

[38] Huang Y, Loux T, Huang X, Feng X (2023). The relationship between chronic diseases and mental health: a cross-sectional study. Ment Health Prev.

[39] Fromer L (2011). Implementing chronic care for COPD: planned visits, care coordination, and patient empowerment for improved outcomes. Int J Chron Obstruct Pulmon Dis.

[40] Hwang M, Zheng Y, Cho Y, Jiang Y (2025). AI applications for chronic condition self-management: scoping review. J Med Internet Res.

[41] Budd G, Griffiths D, Howick J (2022). Empathy in patient-clinician interactions when using telecommunication: a rapid review of the evidence. PEC Innov.

[42] Hemmer P, Schemmer M, Riefle L, Rosellen N, Vössing M, Kühl N Factors that influence the adoption of human-AI collaboration in clinical decision-making. https://aisel.aisnet.org/ecis2022_rp/139/?.

[43] Banerjee S, Alsop P, Jones L, Cardinal RN (2022). Patient and public involvement to build trust in artificial intelligence: a framework, tools, and case studies. Patterns (N Y).

[44] Woodward M, Dixon-Woods M, Randall W (2024). How to co-design a prototype of a clinical practice tool: a framework with practical guidance and a case study. BMJ Qual Saf.

[45] Noorbergen TJ, Adam MTP, Roxburgh M (2021). Co-design in mHealth systems development: insights from a systematic literature review. AIS Trans Hum-Comput Interact.

[46] Osnat B (2025). Patient perspectives on artificial intelligence in healthcare: a global scoping review of benefits, ethical concerns, and implementation strategies. Int J Med Inform.

[47] Prakash S, Balaji JN, Joshi A, Surapaneni KM (2022). Ethical conundrums in the application of artificial intelligence (AI) in healthcare—a scoping review of reviews. J Pers Med.

[48] Weinstein ND (1980). Unrealistic optimism about future life events. J Pers Soc Psychol.

[49] Dumez V, L’Espérance A (2024). Beyond experiential knowledge: a classification of patient knowledge. Soc Theory Health.

