# Supplementary material for: Forecasting the Impacts of Artificial Intelligence Assistance in Virtual Consultations for Chronic Obstructive Pulmonary Disease: Exploratory Futures Wheel Study
Source: J Med Internet Res. 2026 Jun 3;28:e90208. doi: 10.2196/90208 (PMC13233008; doi:10.2196/90208)
Supplement: Multimedia Appendix 1 [file jmir-v28-e90208-s001.docx]

**Futures Wheel workshop topic guide**

*[introduction and Futures Wheel workshop overview]*

Please let’s contemplate the following event:

*“The bespoke AI tool used in every virtual consultation”*

Consider what would be the primary and secondary impacts of this event? Please fill out the Futures Wheel image.

Gather your thoughts around the following impact areas:

- Clinical-patient relationship
- Psychological
- Social
- Educational
- Legal
- Ethical
- Healthcare delivery
- Technological

**Discussion prompts:**

- Consider the ripple effects emerging from the central statement.
- What would the central statement lead to? What would that primary impact lead to?
- There are no right or wrong answers, the future hasn’t been decided, so anything is possible.
- There can be overlaps in your answers.

**Conclusion**

Is there anything else that you would like to add or comment on about the Futures Wheel activity that we haven’t discussed today?

Thank you very much for your time and for your participation today.
